# Myosin II Activity Is Selectively Needed for Migration in Highly Confined Microenvironments in Mature Dendritic Cells

**DOI:** 10.3389/fimmu.2019.00747

**Published:** 2019-04-12

**Authors:** Lucie Barbier, Pablo J. Sáez, Rafaele Attia, Ana-Maria Lennon-Duménil, Ido Lavi, Matthieu Piel, Pablo Vargas

**Affiliations:** ^1^Institut Curie, PSL Research University, CNRS, UMR 144, Paris, France; ^2^Institut Pierre-Gilles de Gennes, PSL Research University, Paris, France; ^3^Université Paris Sud, Université Paris-Saclay, Orsay, France; ^4^Institut Curie, PSL Research University, INSERM U932, Paris, France

**Keywords:** confinement, contractility, chemotaxis, microfabrication, microchannel, collagen

## Abstract

Upon infection, mature dendritic cells (mDCs) migrate from peripheral tissue to lymph nodes (LNs) to activate T lymphocytes and initiate the adaptive immune response. This fast and tightly regulated process is tuned by different microenvironmental factors, such as the physical properties of the tissue. Mechanistically, mDCs migration mostly relies on acto-myosin flow and contractility that depend on non-muscular Myosin IIA (MyoII) activity. However, the specific contribution of this molecular motor for mDCs navigation in complex microenvironments has yet to be fully established. Here, we identified a specific role of MyoII activity in the regulation of mDCs migration in highly confined microenvironments. Using microfluidic systems, we observed that during mDCs chemotaxis in 3D collagen gels under defined CCL21 gradients, MyoII activity was required to sustain their fast speed but not to orientate them toward the chemokine. Indeed, despite the fact that mDCs speed declined, these cells still migrated through the 3D gels, indicating that this molecular motor has a discrete function during their motility in this irregular microenvironment. Consistently, using microchannels of different sizes, we found that MyoII activity was essential to maintain fast cell speed specifically under strong confinement. Analysis of cell motility through micrometric holes further demonstrated that cell contractility facilitated mDCs passage only over very small gaps. Altogether, this work highlights that high contractility acts as an adaptation mechanism exhibited by mDCs to optimize their motility in restricted landscapes. Hence, MyoII activity ultimately facilitates their navigation in highly confined areas of structurally irregular tissues, contributing to the fine-tuning of their homing to LNs to initiate adaptive immune responses.

## Introduction

Antigen delivery from peripheral tissues to LNs by mDCs is critical to initiate the adaptive immune response (1). To ensure its adequacy, this antigen transport needs to occur within a few hours. Consequently, DCs migration to LNs is boosted by signals that trigger their activation, such as pathogen-associated and damage-associated molecular patterns (PAMPs and DAMPs, respectively) (2–5). In this context, we have recently shown that DCs activation leads to a fast and persistent mode of migration, which is linked to the concentration of the acto-myosin cytoskeleton at the cell rear (4–6). MyoII activity generates the force required for mDCs migration in 3D confined microenvironments (7) and is needed for fast and persistent motility during chemotaxis in a dense extracellular matrix (4). Accordingly, failure in inducing MyoII activity is sufficient to delay mDCs homing to draining LNs, with important consequences in the development of immune responses (8).

Importantly, during navigation from the infected tissue to the draining LN, mDCs need to adapt their morphology to the evolving geometrical properties of their microenvironment (9). Recently, several articles have evidenced that distinct cell types increase their MyoII-dependent contractility to migrate in confined microenvironments (10–13). In mesenchymal cells, we have shown that combination of high confinement and low adhesion result in MyoII-dependent fast cell motility *in vitro* (13). In analogy to this observation, fully mature DCs are intrinsically non-adhesive *in vitro* and do not require specific adhesions to migrate in dense 3D microenvironments *in vivo* (7). However, how MyoII activity regulates mDCs motility in response to the degree of confinement remains unexplored.

Here, we combined the use of *ex vivo* imaging and precise *in vitro* microfabricated tools to demonstrate that MyoII activity is important to sustain efficient mDCs navigation exclusively in highly confined microenvironments. Since migratory mDCs possess a high basal level of MyoII activity (6), we propose that this property allows them to adapt their motility to irregular microenvironments found in different tissue compartments. This property might be key to bypass natural physical obstacles in order to reach efficiently the draining LN, ensuring the prompt initiation of the adaptive immune response.

## Inhibition of Cell Contractility Reduces mDCs Migration Speed in a Dense Extracellular Matrix

**Figure 1:**
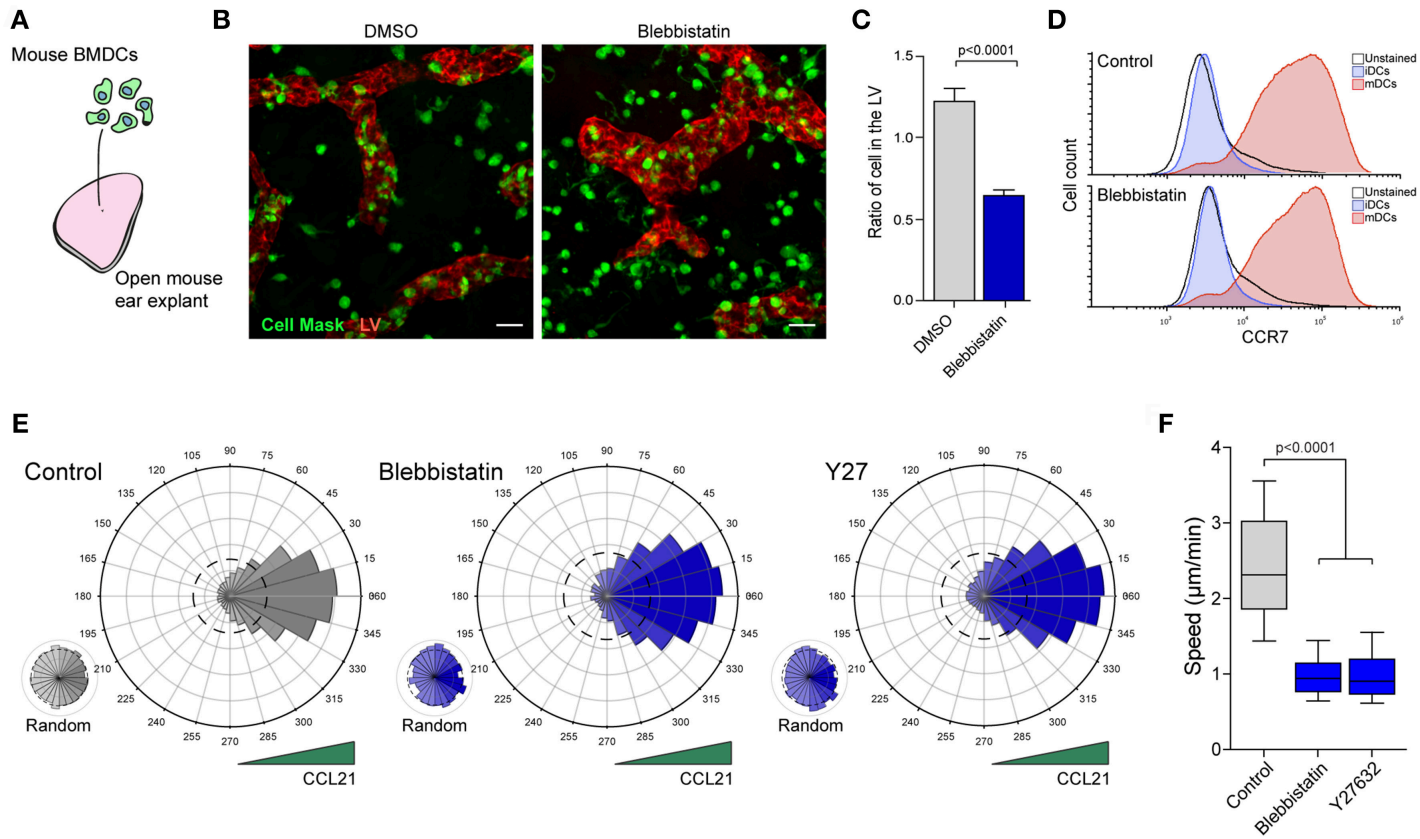
MyoII activity regulates mDCs migration in dense extracellular matrices. **(A–C)** Analysis of mDCs migration in mouse ear explants. **(A)** Schematic representation of the experimental set-up in which *in vitro* differentiated and labeled mDCs were seeded on the dermal side of mouse ear explants. **(B)** Sum z-projection of a representative field from a skin ear explant imaged at 20X on a spinning disk. mDCs are shown in green, LVs stained with anti Lyve-1 in red. Scale bar = 25 μm. **(C)** Quantification of the ratio of mDCs overlapping with the LVs vs. those in the interstitial space. Data from 2 independent experiments, 2 ears explant per experiment and 4 fields of view per explant. Mean and SEM are showed. Unpaired *t*-test with Welch's correction was applied as statistical test. **(D)** Flow cytometry analysis of CCR7 surface expression in mDCs treated or not with Blebbistatin. **(E,F)** Analysis of mDCs trajectories in 3D collagen gels along a CCL21 gradient. **(E)** Polar plots show cell directionality during chemotaxis of control, blebbistatin or Y27632 treated mDCs. Random motility was analyzed in the same gels, but in areas with no access to CCL21. The dashed line in the polar plots indicate the theoretical random motility. One representative experiment out of three is shown (*n* = 323 cells in control, 219 in blebbistatin and 199 in Y27632) **(F)** Mean speed of control, blebbistatin or Y27632 treated mDCs migrating in the directional zone of the gel. Data correspond to the same trajectories as shown in **E**. In the boxplot, the bar and the box include 90 and 75% of the points, respectively. The line inside the box corresponds to the median. The Mann-Whitney test was used as statistical test.

To assess the contribution of MyoII to cell migration in a complex microenvironment, we first used an *ex vivo* model tissue. For that, we evaluated the capacity of exogenous mDCs to reach the LVs in mouse ear explants (4, 14). Briefly, *in vitro* differentiated bone marrow-derived DCs were activated with bacterial lipopolysaccharide (LPS), labeled and seeded in the dermal side of open ear explants either in the absence or presence of the MyoII inhibitor Blebbistatin (Figure 1A). After 1 h of migration, the tissue was fixed and imaged to quantify the number of mDCs that reached the LVs (Figure 1B). Control cells were mostly observed near the lymphatic system or overlapping it, reflecting their strong capacity to migrate toward the LVs. Conversely, in the presence of Blebbistatin, the localization of mDCs was mainly restricted to the area surrounding the LVs (Figure 1B). Accordingly, the ratio of mDCs overlapping the LVs over those remaining in the interstitial space decreased upon MyoII inhibition (Figure 1C). Importantly, these differences were not due to changes in the expression of CCR7, chemokine receptor responsible for driving mDCs migration toward the lymphatic system (Figure 1D). Altogether, these data indicate that MyoII activity is required for the migration of mDCs from the interstitial space toward the LVs in the confined microenvironment of this model tissue.

One limit of this experimental setup is that migration of mDCs in both, the absence or presence of Blebbistatin, cannot be evaluated in the exact same tissue. In addition, despite the short duration of the experiment, the drug might have adverse effects on the tissue itself. Thus, we decided to confirm this result by evaluating the capacity of MyoII conditional knock-out mice mDCs to reach the LVs *ex vivo* (15). For that, exogenous mDCs derived from MyoII-flox/flox/CD11c-Cre- (WT) and MyoII-flox/flox/CD11c-Cre+ (KO) mice were differentially labeled, mixed 50–50% and seeded in the dermal side of the open ear explants (Supplementary Figures 1A,B). In our *in vitro* cultures, we observed a reduction by half in the total amount of MyoII, as measured by western blot (Supplementary Figure 1C). However, this drop was sufficient to reduce significantly the number of mDCs reaching the LVs (Supplementary Figure 1D).

Altogether, these results indicate that MyoII activity is needed for the proper migration of mDCs in ear explants, and a partial decrease in the protein abundance is enough to impair their arrival at LVs, highlighting the relevance of this molecule for mDCs migration in tissues.

Based on these observations, we hypothesized that the decreased mDCs arrival at the lymphatic system might be due to (a) reduced directional migration toward CCL21, the chemokine that guides mDCs toward the lymphatic vessels (14) and/or to (b) a diminished efficiency of mDCs to migrate in the dense extracellular matrix. To test these hypothesis, we used an *in vitro* chemotactic assay in which we assessed the capacity of mDCs to follow a gradient of CCL21 in a dense 3D collagen gel (7, 16). Since MyoII depletion was incomplete in the KO mice, we decided to restrict our experiments to different small inhibitors of this molecular motor. In the control condition, mDCs migrated directionally toward CCL21 (Figure 1E), while in areas of the same gel that were not exposed to the chemokine, their motility remained random (Figure 1E). In Blebbistatin-treated mDCs, directionality toward the chemokine was not affected (Figure 1E, Supplementary Figure 1E and Supplementary Movie 1) while cell speed was markedly reduced (Figure 1F). Slower cell migration was also observed during random motility, indicating that MyoII activity is required for fast cell migration in the 3D extracellular matrix, but is dispensable for sensing or orientation of mDCs toward CCL21 (Supplementary Figure 1F). Similar results were obtained from inhibiting the rho-activated kinase (ROCK) using Y27632 (Y27), which also leads to decreased MyoII activity (Figures 1E,F, Supplementary Figure 1F). These results indicate that MyoII inhibition causes a strong decrease in mDCs migration in 3D microenvironments.

## Myosin II Is Required for mDCs Migration in Highly Confined Microenvironments

A striking property of 3D collagen gels is the geometrical irregularity imposed to cells, which forces them to transit through zones of variable confinement. Interestingly, inhibition of MyoII activity in mDCs reduces their migration speed in the 3D gel, but without stopping completely their movement (Supplementary Movie 1). Based on this observation, we hypothesized that MyoII activity could influence specific steps of mDCs motility depending on the degree of confinement encountered in irregular 3D landscapes.

**Figure 2:**
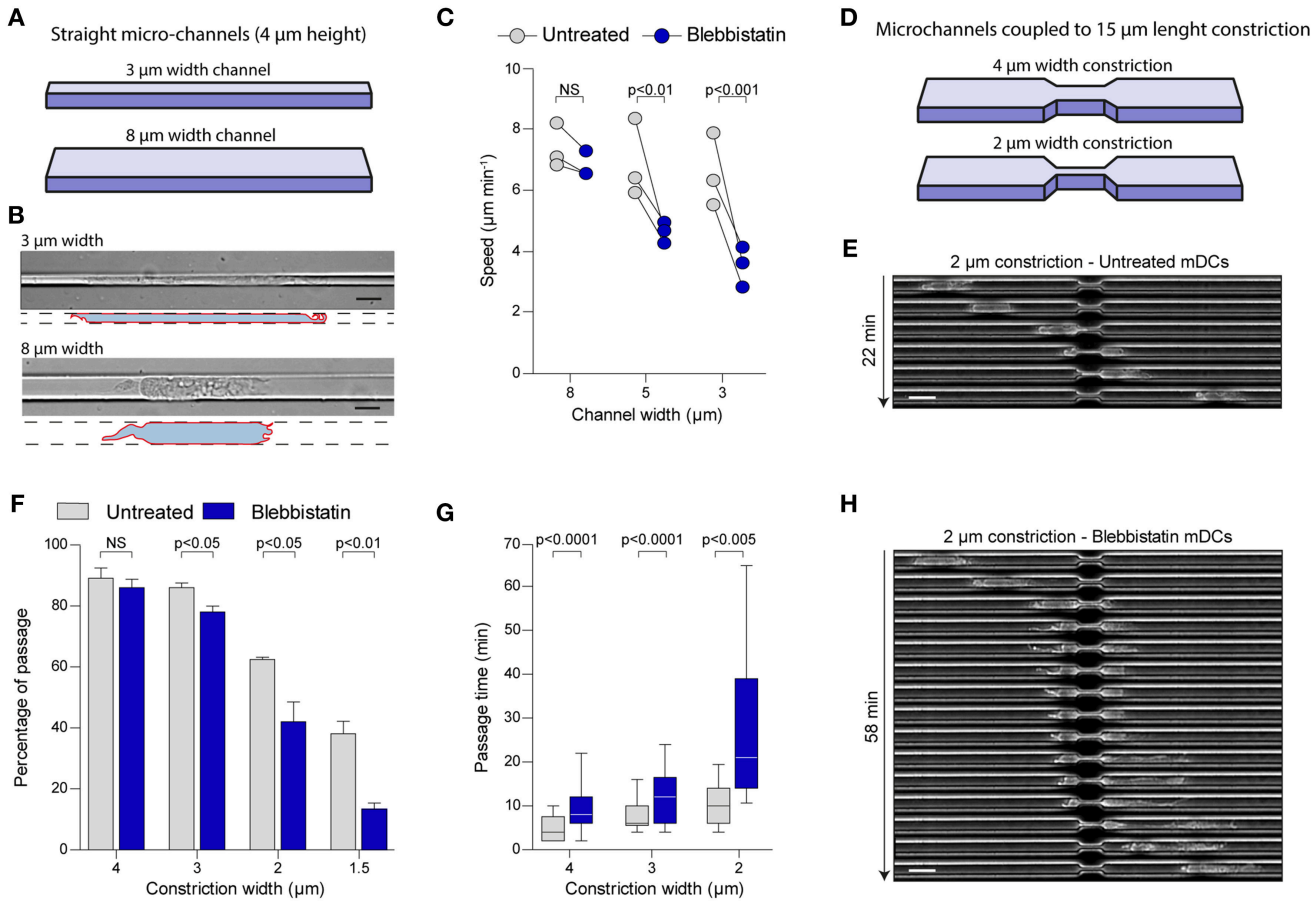
MyoII motor activity is specifically required for mDCs migration in very confined microenvironments. **(A–C)** Analysis of mDCs migration in straight microchannel of different width. **(A)** Schematic representation of the microchannel used, where the height of the microchannel is fixed at 4 μm, and the width of the channel vary from 3 to 8 μm. **(B)** Representative mDCs in 3 and 8 μm width microchannel imaged at 40X with DIC. The drawing highlights the contour of the cells. Scale bar = 10 μm. **(C)** Mean instantaneous speed of untreated or blebbistatin treated mDCs in microchannel of 3, 5, and 8 μm width obtained in three independent experiments. Each dot represents the median of one experiment (*n* > 30 cells for each condition in each experiment). Anova with Tukey's Multiple Comparison Test was applied as statistical test. **(D–H)** Analysis of mDCs passage through constrictions of different sizes. **(D)** Schematic representation of the constrictions added in the microchannel, the height and the length of the constriction are fixed to 4 and 15 μm, respectively, the width of the constriction varies from 1.5 to 4 μm. **(E)** Sequential image of a mDCs passing through a 2 μm width constriction acquired with phase contrast and a 10X objective. Scale bar = 20 μm. **(F)** Percentage of the untreated and treated mDCs passing through the first constriction of the chamber amongst all cells touching it. Bars represent mean and SEM from three independent experiments (*n* > 95 cells for each condition in each experiment). Unpaired *t*-test was applied as statistical test. **(G)** Time spent in the constriction by mDCs passing the constriction. *N* = 72, 142, and 156 untreated mDCs in 2, 3, and 4 μm width constrictions; *n* = 42, 70, and 142 for blebbistatin treated mDCs in 2, 3, and 4 μm width constrictions. The bar and the box include, respectively, 90 and 75% of the points, the center corresponds to the median. One representative experiment out of three is shown. Unpaired *t*-test with Welch's correction was applied for statistical test. **(H)** Sequential image of a blebbistatin treated mDCs passing through a 2 μm width constriction acquired with phase contrast and a 10X objective. Scale bar = 20 μm.

To test this idea, we took advantage of the precise and diverse geometries that can be generated by using microfabrication (17, 18). With this technology, we designed microchannels of different sizes (8, 5, or 3 μm width by 4 μm height) to challenge cells to migrate in microenvironments with increasing degrees of confinement (Figure 2A). First, we observed that mDCs were able to migrate spontaneously independently of the confinement level. Strikingly, despite a strong effect on the cell shape due to the confinement in small microchannels (i.e., 3 μm) (Figure 2B), mDCs speed was only slightly reduced in these tubes (Figure 2C, Supplementary Figure 2A). This suggests that mDCs activate a specific cellular machinery to maintain efficient migration in very confined geometries. Surprisingly, mDCs treatment with Blebbistatin did not significantly affect their speeds in larger channels (i.e., 8 μm) (Figure 2C, Supplementary Figure 2A, and Supplementary Movie 2), but decreased them only under higher confinement (30 and 50% speed reduction in 5 and 3 μm channels, respectively) (Figure 2C, Supplementary Figure 2A, and Supplementary Movie 2). Similar results were obtained from using the ROCK inhibitor Y27, indicative of the phenotype robustness (Supplementary Figure 2B). For us, the simplest interpretation of this result is that large channels impose little resistance to migration, and thus the force provided by MyoII is not needed. In contrast, MyoII becomes critical to maintain cell speed in small channels, which impose more resistance to their motility. These results suggest that mDCs regulate MyoII activity depending on the degree of confinement.

## MyoII Activity Is Required for mDCs Passage Through Small Gaps

The constant confinement provided by straight microchannels does not recapitulates the complex geometry of dense extracellular matrices, which display multiple irregularities (19). Thus, we decided to study mDCs migration in microchannels coupled to micrometric constrictions, to evaluate the precise contribution of MyoII activity to migration through an irregular landscape (Figure 2D) (9, 16). For that, we used 8 μm width microchannels and added constrictions of different sizes, ranging from 4 to 1.5 μm width, over 15 μm length (Figure 2D) (9). Since in these channels MyoII inhibition does not affect cell speed (Figure 2B and Supplementary Figure 2A), this system allowed us to evaluate the specific role of cell contractility in the passage of mDCs through small pores (Figure 2E). First, we evaluated the percentage of cells effectively passing through the different constrictions (Figure 2F). Our experiments showed that in control cells, more than 80% of the mDCs passed through 4 and 3 μm width constrictions, while 60 and 40% of them were able to overcome 2 and 1.5 μm pores, respectively (Figure 2F). No significant effect was observed on the fraction of cells migrating through 4 μm width constrictions upon MyoII inhibition with Blebbistatin (Figure 2F). However, the drug impact intensified progressively with the constriction narrowing, ending up with a 75% inhibition of the passage through the smallest pores (1.5 μm width) (Figure 2F). A similar effect was induced by the ROCK inhibitor Y27 (Supplementary Figure 2C). Altogether, these data indicate that MyoII activity in mDCs is needed exclusively to pass through narrow gaps smaller than 3 μm in width.

Next, as an indicator of the cell efficiency in deforming and passing through the small gaps, we calculated the time spent by each cell inside a constriction, only if they succeeded to migrate through it. In the analysis, we observed that this time also increased with the pore narrowing, starting at 5 min in average for the 4 μm constriction, and ending with 10 min when moving through the 2 μm gaps (Figure 2G). These observations indicate that unlike straight microchannels, the pore size of an irregular microenvironment can be determinant to restrict mDCs migration. In this setup, MyoII inhibition using blebbistatin systematically increased the time spent by mDCs in the constriction as compared to the control condition, independently of gap size (Figures 2G,E,H). Similarly, ROCK inhibition also doubled the passage time of cells through the constrictions for all pore sizes (Supplementary Figure 2D). Of note, due to the low proportion of mDCs able to pass through the 1.5 μm constrictions upon MyoII inhibition, the passage time was not considered for this specific condition.

Collectively, these data indicate a dual role of MyoII activity in the migration of mDCs through small gaps: (i) it is required for cell passage through very small holes and (ii) to maintain cell speed while squeezing and deforming in an irregular landscape. Combined with the data obtained from collagen gels and straight microchannels, our experiments demonstrate that MyoII activity in mDCs helps them to adapt their migration to irregular and restrictive microenvironments. This suggests a specific adaptation of mDCs that ensures fast homing from infected tissues to LNs, a situation that imposes a series of physical constrains as cells translocate between distant locations in the organism.

## Discussion

Migration from peripheral tissues to lymph nodes is a challenging function for mDCs, which must constantly move through different body compartments adapting to the changing properties of the tissues. Here, we found that MyoII activity in mDCs is required specifically to maintain their speed and squeeze through highly confined microenvironments. In ear explants, MyoII inhibition impaired mDCs arrival at the lymphatic system, which was not due to a defect in the detection of CCL21 gradient but rather to a defect in their intrinsic motile capacity, as measured in collagen gels. This is in agreement with data showing that cell speed can be dissociated from directionality during chemotaxis, having as consequence a decrease in the quality of cell migration *in vitro* and *ex vivo* (4, 20, 21). Interestingly, partial depletion of MyoII was enough to decrease cell migration of mDCs in ear explants. Recently, similar observations were obtained in neutrophils, in which partial depletion of MyoII diminished migration in confinement (22). Together with our data, these evidences indicate that motility of mDCs and neutrophils in confinement is highly sensitive to the levels or activity of MyoII.

Interestingly, inhibition of MyoII reduces the speed, but does not fully stops cells as they migrate in a collagen gel. Cell speed during random motility and chemotaxis decreases in both cases, but cells are still able to move. Since the microenvironment in a 3D gel is irregular, this can be explained by a specific role of MyoII during migration in the more restricted areas of the gel. In agreement with this idea, recent articles have shown MyoII activation under compression (10–13).

Surprisingly, in our experiments, migration speed in large channels was not affected by MyoII inhibition. One possibility to explain this result is that MyoII is simply not required for their movement in such a microenvironment. However, in previous studies obtained by our group, we have seen that MyoII inhibition triggers a global reorganization of the actin cytoskeleton in mDCs when migrating in large channels (6). This suggests the existence of distinct modes of motility that can operate in these cells, resulting in both cases in fast migration *in vitro* when confinement is not strong. Interestingly, mDCs have been previously shown to adapt their motility to the adhesive properties of their microenvironment, alternating distinct modes of migration that sustain fast speed (23). Altogether, these observations indicate the existence of different types of migratory machineries in mDCs that depending on the properties of their microenvironment can compensate to ensure their migratory function.

Migration under strong confinement (small channels and constrictions) required MyoII activity. This indicates that despite the existence of different modes of motility in mDCs, extreme confinement needs a specific migratory mechanism that relies on cell contractility. This mechanism also applies for mDCs migration in dense collagen gels, showing that this need is maintained during migration in more complex landscapes (7). This adaptation is not universal, since some tumor cells and human mesenchymal stem cells have been shown to use a contractility-independent mode of motility under confinement (24, 25). Interestingly, the MyoII requirement to migrate in small holes seems specific to mDCs, since we have shown that passage of immature DCs through constrictions was independent of cell contractility and required Arp2/3-mediated actin nucleation (9). These differences might result in an additional level of control for the migration of mDCs, in which the regulation of MyoII contractility by inflammatory or environmental factors might participate in the fine tuning of their migration to LNs. This MyoII-dependency might be a global requirement for leukocyte migration between tissues, since neutrophils and T lymphocytes also require contractility to squeeze through confined landscapes (13, 26).

Our study also showed that the geometry of the microenvironment has a strong impact on mDCs migration, especially in irregular spaces where the size of the pores limits mDCs passage. Thus, modification in the density of the extracellular matrix may also modulate mDCs arrival at LNs. This property can be particularly relevant during mDCs migration through distinct organs, which display intrinsic differences in their stiffness (27) or during inflammation, known to alter the physical properties of the tissue (28). The same principle can apply during cancer progression, that often alter the properties of the extracellular matrices surrounding the core of the tumor (29, 30) and might prevent immune cells infiltration (31). Thus, modulation of contractility could be used as a general approach to optimize mDCs motility in pathological conditions.

Importantly, the behavior of mDCs relative to the different degrees of confinement suggests a mechano-response in these cells. However, the mechanism(s) that sense the geometry of the tissue and adapt MyoII activity remain unknown. A local control of mDCs contractility has been already reported to promote their transmigration, where MyoII activity was modulated by chemical signals from lymphatic endothelial cells (32). Also, a possible specific regulatory mechanism might come from lysosomal signaling, that we have recently shown to regulate MyoII-induced contractility at the back of mDCs (6). Understanding how MyoII activity is modulated in response to confinement might provide molecular tools to modulate cell migration through specific tissues. In particular, in inflammatory diseases such as auto-immune encephalopathies or allergic contact dermatitis, down regulation of MyoII activity prevents mDCs migration to LNs and limits inflammation (8), while in other situations, such as tumors, increasing their migration might be beneficial (33). A better understanding of the mechanisms regulating mDCs contractility under confinement can create new routes to the development of molecules to tune the adaptive immune response with therapeutic purposes.

## Methods

### Cells

Bone marrow derived dendritic cells (BMDCs) were obtain by differentiation of bone morrow precursors for 10 days in DCs medium (IMDM-Glutamax, FCS 10%, pen-strep 100 U ml^−1^, and 2-ME 50 μM) supplemented with granulocyte-macrophage colony stimulating factor (GM-CSF)-containing supernatant (50 ng ml^−1^) obtained from transfected J558 cell line, as previously described (34). Briefly, at day 10 of differentiation, semi-adherent DCs were treated with LPS (100 ng ml^−1^) for 30 min, then washed 3 times with DCs medium and cultured overnight (ON) to reach full DC maturation. Migration of mDCs was recorded between 24 and 34 h post LPS treatment.

### Mice

BMDCs were obtained from wild-type C57BL/B6 mice (Charles River). In the case of MyoII KO, BMDCs were differentiated from MyoIIA-flox/flox-CD11c-Cre+ mice, as previously described (15, 35, 36). Littermate MyoIIA-flox/flox CD11c-Cre- were used as a control. In general, 6 to 10 weeks old mice were used as source for bone marrows; 4 to 6 weeks old mice were used as ear explant donors. For animal care, we strictly followed the European and French National Regulation for the Protection of Vertebrate Animals used for Experimental and other Scientific Purposes (Directive 2010/63; French Decree 2013-118). The present experiments, which used mouse strains displaying non-harmful phenotypes, did not require a project authorization and benefited from guidance of the Animal Welfare Body, Research Centre, Institut Curie.

### Antibodies and Reagents

For drug treatment, Blebbistatin (50 μM, Sigma) and the equivalent amount of DMSO (Sigma-Aldrich), or Y-27632 (10 μM, Tocris Bioscience) and the equivalent amount of distillated water were used. For labeling of lymphatic vessels in mouse era explants, Alexa Fluor 655-coupled anti-Lyve-1 antibody was used (R&D System, 1/50). To label BMDCs for migration in ear explants we used Hoechst 33342 (200 ng ml^−1^, Life Technologies) and CellMask CFSE or CMTMR (2.5 μM) (Life Technologies). For western blot, Non-muscle Myosin Heavy Chain II-A Antibody (Biolegend, clone Ply19098, 1/200) and GAPDH Antibody (Cell Signaling, clone 14c10, 1/5,000) were used. For flow cytometry analysis: Mouse CCL19-Fc Fusion Recombinant Protein (1/400, eBioscience) and Alexa-Fluor 488-coupled anti-human Fc (1/400, eBioscience).

### Migration in Ear Explant

Migration of DCs was performed as previously described (14) but modified to work with fixed samples. Briefly, ears were excised from C57BL/6 mice and the ventral part of the skin was peeled off to expose their dermal side. 100,000 colored LPS-activated BMDCs were added on the top of the exposed dermal side of the skin explant. After 1 h of incubation, explants were washed to remove the loosely attached mDCs and fixed during 20 min on a drop of 4% paraformaldehyde (Sigma-Aldrich). In the case of Blebbistatin treatment, the cells were colored and pre-incubated 2 h with the drug before seeding in the ear and was further maintained during migration in the explants. In the case of MyoII WT and KO mDCs, 75,000 colored LPS-MyoII KO BMDCs were mixed with 75,000 colored LPS-MyoII WT BMDCs and added on the top of the exposed dermal side of the skin explant. After fixation, the explants were washed in PBS 2%-BSA and the lymphatic vessels were stained with Alexa Fluor 655-coupled anti-Lyve-1 antibody 1 h at 4°C. After three washes, skin explants were mounted in a microscopy slide using fluoromount-G (Invitrogen) and imaged on a Spinning disk confocal CSU X1 inverted microscope (Leica) and a × 20 dry objective (NA 0.75). For Blebbistatin experiments, mDCs overlapping or not with the lymphatic system were manually count on a SUM z-projection. For MyoII-KO experiments, a custom ImageJ macro was used to count the number of nucleus corresponding to each phenotype. Briefly, a SUM z-projection was made and, using appropriated thresholding, we detected MyoII-WT and MyoII-KO nucleus. Then, using a mask obtained from the lymphatic vessels, we counted the numbers of nucleus of MyoII-WT and MyoII-KO mDCs overlapping or not with the lymphatic system. The ratio of mDCs in the lymphatic vessels was calculated as the number of nucleus in the lymphatic vessel divided by the number of nucleus outside the lymphatic vessels.

### Migration in Collagen Gels

Collagen experiments were performed as previously described (16). Briefly, mDCs were mixed at 4°C with rat tail collagen type I (Corning) at 3 mg ml^−1^ at basic pH and loaded in the custom-made chamber in polydimethylsiloxane (PDMS). The sample was incubated at 37°C for 30 min to allow gel polymerization. Then, 2 ml of DC medium containing 200 ng ml^−1^ CCL21 (R&D Systems) was added in the dish, generating a chemokine gradient that triggered directed mDC migration. When indicated, cells were pre-incubated 1 h with blebbistatin at 50 μM or Y27 at 10 μM, and then maintained in the media during their chemotaxis. Cells were imaged overnight with a DMi8 inverted microscope (Leica) at 37 °C with 5% CO_2_ atmosphere and a × 10 dry objective (NA 0.40 phase). Resulting movies were processed with average subtraction, mean filter and Gaussian Blur filter to obtain cells as white round object on a dark background. Tracking was performed with Imaris software in the first 400 μm from the border of the chamber, where the gradient is stable. Tracks of objects moving <10 μm length or lasting <10 min were removed from the analysis to avoid artifacts.

### Migration in Micro-Channels

Micro-channels experiments were performed as previously described (18). Briefly, PDMS (RTV615, Neyco) was used to make microchannels of the different geometries from custom-made molds. The micro-channels were coated with bovine plasma fibronectin (10 μg ml^−1^) (Sigma-Aldrich) for 1 h at RT and washed 3 times with PBS before incubating with DC medium for at least 1 h at 37°C and 5% CO_2_ before cell loading. When indicated, this media also contained 50 μM blebbistatin or 10 μM Y27632. Migrating cells were recorded overnight with a DMi8 inverted microscope (Leica) at 37 °C with 5% CO_2_ atmosphere and a × 10 dry objective (NA 0.40 phase). One image every 2 min during 16 h was recorded.

### Quantification of Cell Migration in Micro-Channels

Kymographs for each channel were generated using a semi-automated ImageJ macro. For velocity measurements, kymographs from isolated migrating cells were manually extracted and analyzed using a custom Matlab program as previously described (34). For cell passage through constrictions, kymographs from each channel were analyzed using a semi-automated ImageJ macro. We focused on the passage of the first constriction encountered by the cell. The percentage of passage represents the ratio between the number of cells that passed a constriction respect to the number of cells that encountered a constriction. The passage time represents the time between the time at which the cell front reaches the constriction and the time at which the cell back exits the constriction.

### Immunobloting

1.5 millions of mDCs were lysed for 30 min in 40 μl of lysis buffer containing 100 mM Tris, 150 mM NaCl, 0.5% NP-40 and a protease inhibitor cocktail tablet (Roche). 10 μl of extracts were loaded onto a 4–20% TGX gradient gel (BioRad) and transferred onto an Ethanol-activated PVDF membrane by over-night wet transfer (BioRad). The membrane was blocked, incubated sequentially with the appropriate antibodies and revealed using the SuperSignal West Pico Chemiluminescent substrate (Thermo Scientific). Membranes were cut accordingly to the molecular weight of the protein of interest. This allowed us to evaluate different labeling in the same run. As consequence, full membranes were in most cases only fragments.

### Flow Cytometry Analysis

750,000 mDCs pre-incubated 2 h with Blebbistaitin or DMSO as a control were resuspended in 50 μl of PBS 2% BSA alone or with Mouse CCL19-Fc Fusion Recombinant Protein. After 1 h of staining at 4°C, cells were washed two times and incubated for 1 h with Alexa Fluor 488-coupled anti-human Fc at room temperature. After two washes, cells were resuspended in 200 μl of PBS 2% BSA. Single cell fluorescence were measured on a Accuri flow cytometer and analyses with FCS Express 6 software. Appropriated gating was made on the SSC/FFC signal.

## Author Contributions

LB performed and analyzed most experiments, prepared manuscript figures and strongly participated in article writing. PS performed and analyzed collagen gels experiments and contributed to article writing. RA designed the microchannels and performed photolithography. A-ML-D provided animal models and contributed to article correction. IL wrote the code to analyze cell trajectories in collagen gels. MP participated in experiment design and contributed to article writing. PV designed the overall research and wrote the manuscript.

### Conflict of Interest Statement

The authors declare that the research was conducted in the absence of any commercial or financial relationships that could be construed as a potential conflict of interest.

## Supplementary Material

Supplementary Figure 1 **(A,B)** Analysis of mDCs migration in mouse ear explants. **(A)** Schematic representation of the experimental set-up in which *in vitro* differentiated and labeled mDCs were seeded on the dermal side of mouse ear explants. **(B)** Sum z-projection of a representative field from a skin ear explant imaged at 20X on a spinning disk. mDCs are shown in green, LVs stained with anti Lyve-1 in white. Scale bar = 30 μm. **(C)** Quantification of the ratio of mDCs overlapping with the LVs vs. those in the interstitial space. Data from two independent experiments, three ears explant per experiment and three field of view per explant. Mean and SEM are showed. Paired *t*-test was used as statistical test. **(D)** Western blot analysis of Myosin II A heavy chain expression in mDCs derived from MyoII-flox/flox/CD11c-Cre- (WT) and MyoII-flox/flox/CD11c-Cre+ (KO) mice. Histogram shows the quantification of the plot. **(E)** Directionality index of mDCs migrating in collagen gels. Gray bars correspond to random trajectories and blue bars to tracks during chemotaxis. This analysis is based on the data shown in Figure 1E. **(F)** Representative experiment showing the mean speed of control, blebbistatin or Y27632 treated mDCs migrating randomly in a collagen gel. Control *n* = 341, blebbistatin *n* = 104, Y27632 *n* = 90. Three independent experiments were performed. In the boxplot, the bar and the box include 90 and 75% of the points, respectively. The line inside the box corresponds to the median. The Mann-Whitney test was used as statistical test.

Supplementary Figure 2 **(A)** Representative experiment of mDCs migrating in microchannels of different sizes. The figure shows the mean instantaneous speed of untreated or blebbistatin treated mDCs in microchannel of 3, 5, and 8 μm width. *N* = 91, 109, and 178 untreated mDCs in 8, 5, and 3 μm width microchannel, respectively; *n* = 53, 85 and 66 for blebbistatin treated mDCs in 8, 5, and 3 μm width microchannels, respectively. Unpaired t-test was applied as statistical test with Welch's correction for 3 μm width microchannel. **(B)** Mean instantaneous speed of untreated or Y27632 treated mDCs in microchannel of 3, 5, and 8 μm width obtained in three independent experiments. Each dot represents the median of one experiment (*n* > 30 for each condition in each experiment). Anova with Tukey's Multiple Comparison Test was applied as statistical test. **(C)** Percentage of untreated and Y27632 treated mDCs passing through the first constriction of the chamber amongst all cells touching it. One experiment with *n* = 76, 54, 105, and 111 untreated mDCs in 1.5, 2, 3, and 4 μm width constrictions; *n* = 53, 64, 122, and 107 for Y-27632 treated mDCs in 1.5, 2, 3, and 4 μm width constrictions **(D)** Time spent in the constriction by mDCs passing the constriction in the presence or absence of Y27632. The bar and the box include respectively 90 and 75% of the points, the center corresponds to the median. One experiment with *n* = 69, 91, and 100 untreated mDCs in 2, 3, and 4 μm width constrictions; *n* = 32, 104, and 88 for Y-27632 treated mDCs in 2, 3, and 4 μm width constrictions. Unpaired *t*-test was applied for statistical test.

Supplementary Movie 1 MyoII inhibition decreases cells speed of mDCs during chemotaxis in dense collagen gels. mDCs activated with LPS were seeded in 3 mg/ml rat tail collagen gels and exposed to 200 ng/ml CCL21. Control and blebbistatin treated cells are able to follow the chemokine gradient. Despite decreased speed, MyoII inhibition does not impact cell directionality. Bar = 100 μm.

Supplementary Movie 2 MyoII inhibition decreases cells speed of mDCs exclusively in the 3 μm-width microchannels. The movie shows mDCs spontaneously migrating in 8 μm **(left)** or 3 μm **(right)** width microchannels. MyoII inhibition using Blebbistatin decreases mDCs speed only in the 3 μm width microchannel. Image acquired with phase contrast and a 10X objective, one image every 2 min, time indicated in h:min.
