# Supplementary figures and images for: Myosin II Activity Is Selectively Needed for Migration in Highly Confined Microenvironments in Mature Dendritic Cells
Source: Front Immunol. 2019 Apr 12;10:747. doi: 10.3389/fimmu.2019.00747 (PMC6474329; doi:10.3389/fimmu.2019.00747)

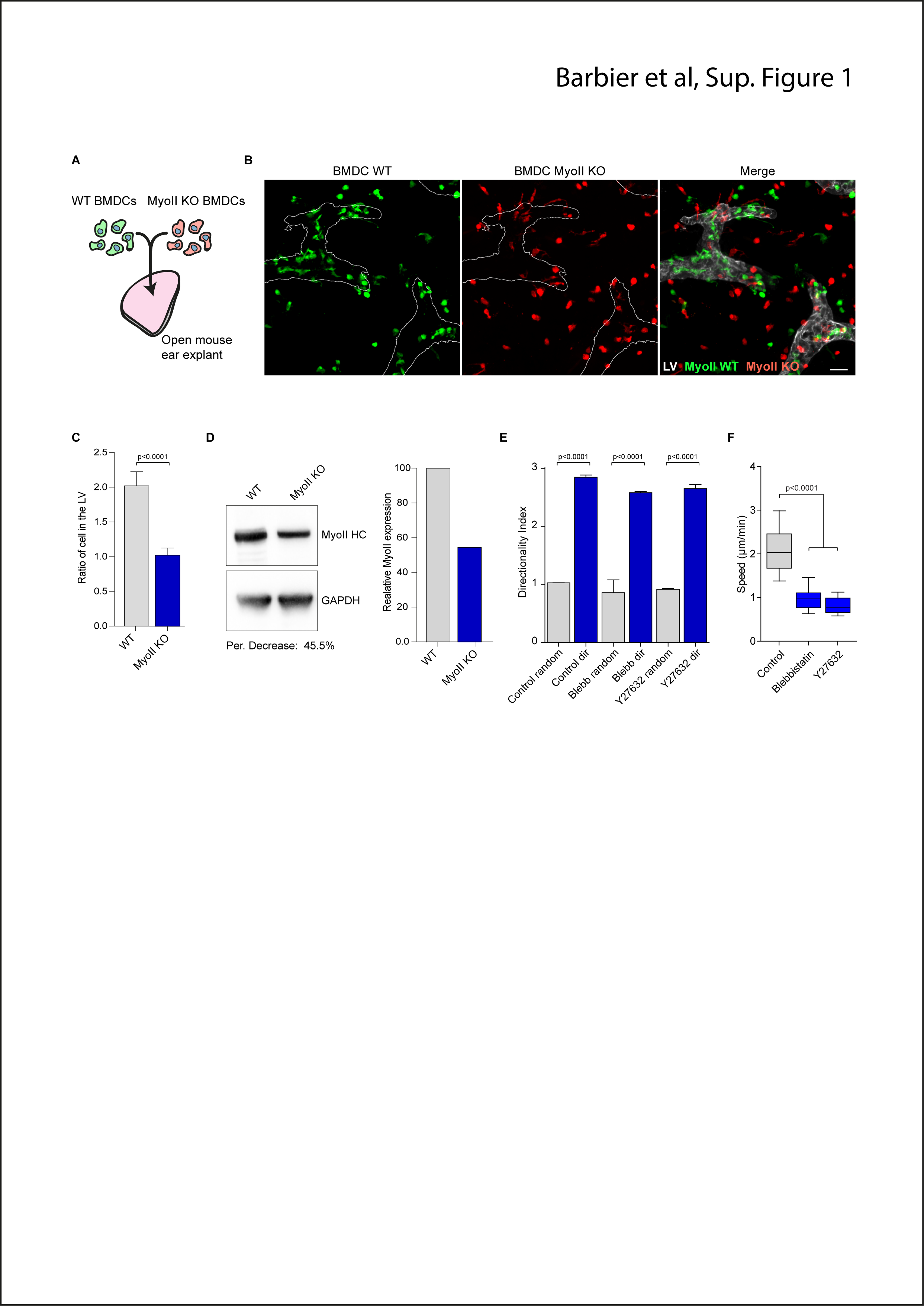

Supplement: Supplementary Figure 1 — (A,B) Analysis of mDCs migration in mouse ear explants. (A) Schematic representation of the experimental set-up in which in vitro differentiated and labeled mDCs were seeded on the dermal side of mouse ear explants. (B) Sum z-projection of a representative field from a skin ear explant imaged at 20X on a spinning disk. mDCs are shown in green, LVs stained with anti Lyve-1 in white. Scale bar = 30 μm. (C) Quantification of the ratio of mDCs overlapping with the LVs vs. those in the interstitial space. Data from two independent experiments, three ears explant per experiment and three field of view per explant. Mean and SEM are showed. Paired t-test was used as statistical test. (D) Western blot analysis of Myosin II A heavy chain expression in mDCs derived from MyoII-flox/flox/CD11c-Cre- (WT) and MyoII-flox/flox/CD11c-Cre+ (KO) mice. Histogram shows the quantification of the plot. (E) Directionality index of mDCs migrating in collagen gels. Gray bars correspond to random trajectories and blue bars to tracks during chemotaxis. This analysis is based on the data shown in Figure 1E. (F) Representative experiment showing the mean speed of control, blebbistatin or Y27632 treated mDCs migrating randomly in a collagen gel. Control n = 341, blebbistatin n = 104, Y27632 n = 90. Three independent experiments were performed. In the boxplot, the bar and the box include 90 and 75% of the points, respectively. The line inside the box corresponds to the median. The Mann-Whitney test was used as statistical test. [file Image_1.TIF]

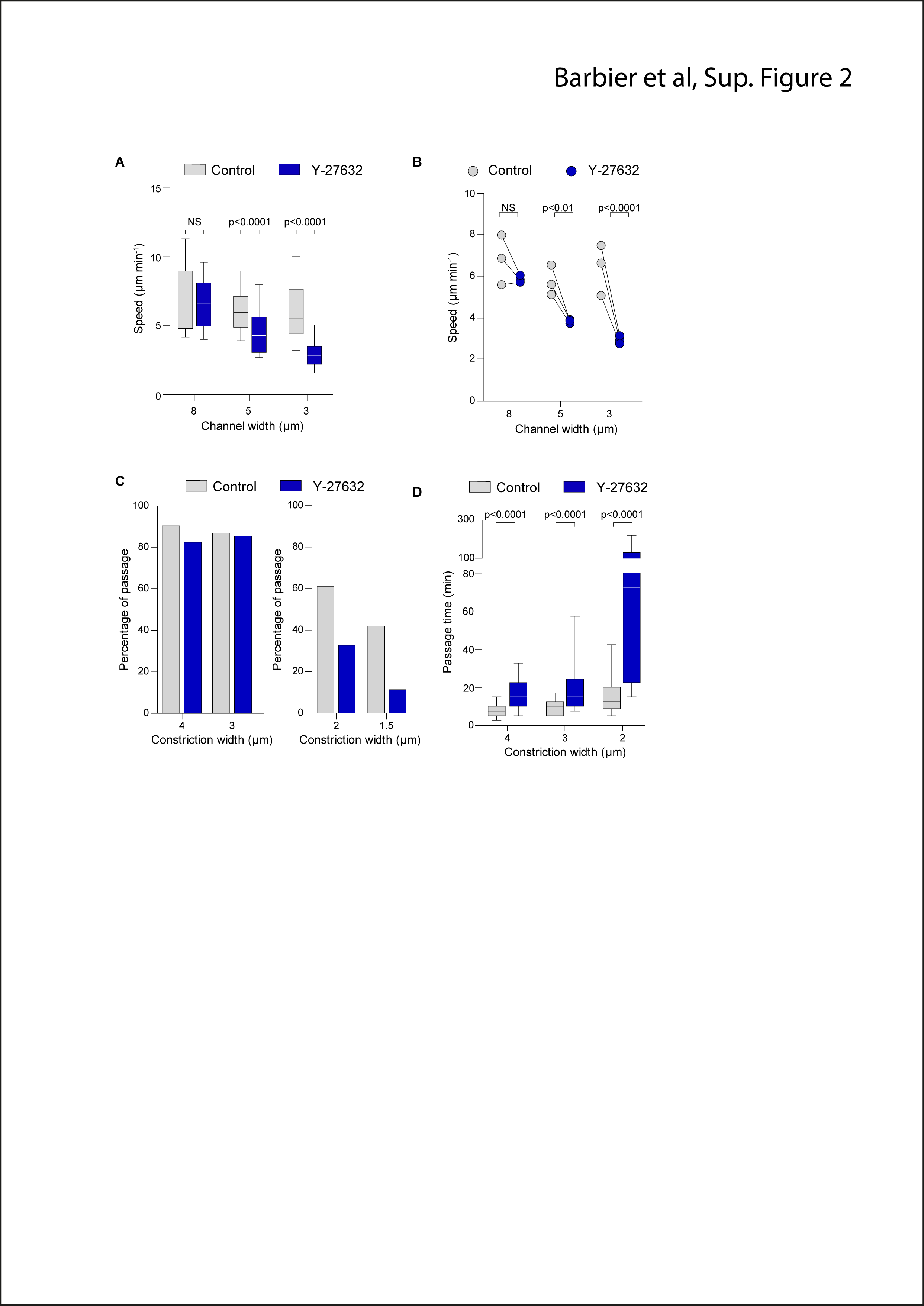

Supplement: Supplementary Figure 2 — (A) Representative experiment of mDCs migrating in microchannels of different sizes. The figure shows the mean instantaneous speed of untreated or blebbistatin treated mDCs in microchannel of 3, 5, and 8 μm width. N = 91, 109, and 178 untreated mDCs in 8, 5, and 3 μm width microchannel, respectively; n = 53, 85 and 66 for blebbistatin treated mDCs in 8, 5, and 3 μm width microchannels, respectively. Unpaired t-test was applied as statistical test with Welch's correction for 3 μm width microchannel. (B) Mean instantaneous speed of untreated or Y27632 treated mDCs in microchannel of 3, 5, and 8 μm width obtained in three independent experiments. Each dot represents the median of one experiment (n > 30 for each condition in each experiment). Anova with Tukey's Multiple Comparison Test was applied as statistical test. (C) Percentage of untreated and Y27632 treated mDCs passing through the first constriction of the chamber amongst all cells touching it. One experiment with n = 76, 54, 105, and 111 untreated mDCs in 1.5, 2, 3, and 4 μm width constrictions; n = 53, 64, 122, and 107 for Y-27632 treated mDCs in 1.5, 2, 3, and 4 μm width constrictions (D) Time spent in the constriction by mDCs passing the constriction in the presence or absence of Y27632. The bar and the box include respectively 90 and 75% of the points, the center corresponds to the median. One experiment with n = 69, 91, and 100 untreated mDCs in 2, 3, and 4 μm width constrictions; n = 32, 104, and 88 for Y-27632 treated mDCs in 2, 3, and 4 μm width constrictions. Unpaired t-test was applied for statistical test. [file Image_2.TIF]
